# Hidden Addressing Encoding for DNA Storage

**DOI:** 10.3389/fbioe.2022.916615

**Published:** 2022-07-19

**Authors:** Penghao Wang, Ziniu Mu, Lijun Sun, Shuqing Si, Bin Wang

**Affiliations:** The Key Laboratory of Advanced Design and Intelligent Computing, Ministry of Education, School of Software Engineering, Dalian University, Dalian, China

**Keywords:** DNA storage, DNA encoding, random access, hidden addressing, encoding sequence local performance, index overall self-similarity

## Abstract

DNA is a natural storage medium with the advantages of high storage density and long service life compared with traditional media. DNA storage can meet the current storage requirements for massive data. Owing to the limitations of the DNA storage technology, the data need to be converted into short DNA sequences for storage. However, in the process, a large amount of physical redundancy will be generated to index short DNA sequences. To reduce redundancy, this study proposes a DNA storage encoding scheme with hidden addressing. Using the improved fountain encoding scheme, the index replaces part of the data to realize hidden addresses, and then, a 10.1 MB file is encoded with the hidden addressing. First, the Dottup dot plot generator and the Jaccard similarity coefficient analyze the overall self-similarity of the encoding sequence index, and then the sequence fragments of GC content are used to verify the performance of this scheme. The final results show that the encoding scheme indexes with overall lower self-similarity, and the local thermodynamic properties of the sequence are better. The hidden addressing encoding scheme proposed can not only improve the utilization of bases but also ensure the correct rate of DNA storage during the sequencing and decoding processes.

## 1 Introduction

With the rapid development of information technologies such as the Internet and artificial intelligence, the amount of global information has exploded. In the future, the amount of global data will soon exceed the storage capacity of the current storage media. Therefore, a high-capacity storage medium is urgently needed to store a large amount of data. DNA data storage is a new storage method that can play an important role in saving storage energy and promoting the development of data storage (Newman et al., 2019; Choi et al., 2020; Dong et al., 2020). DNA is a natural information storage medium with high data storage density, long storage time, and low loss rate (Chen et al., 2019; Matange et al., 2021). In the aspects in which the traditional storage methods cannot meet the information needs, DNA data storage has gradually become a popular topic in the research field of biological information (Ceze et al., 2019; Xu et al., 2021).

**FIGURE 1:**
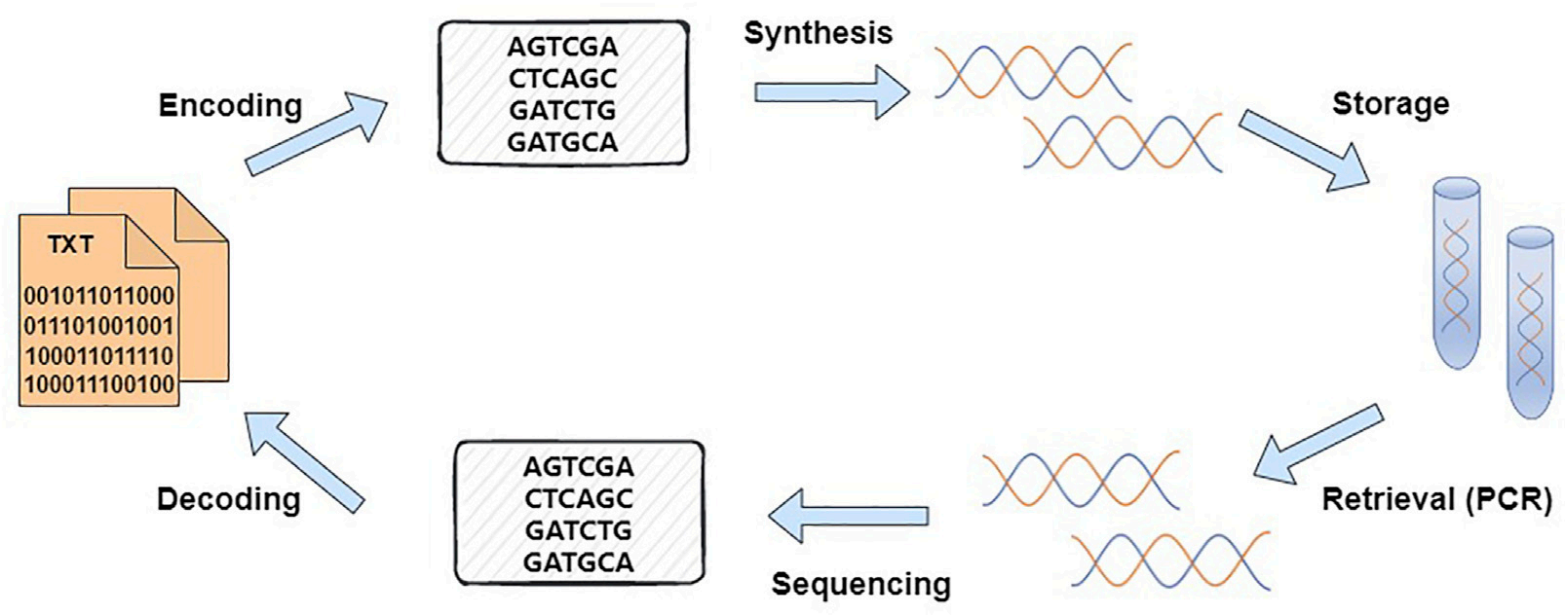
Overall schematic of DNA storage.

The basic process of DNA data storage comprises four main steps: encoding, synthesis, sequencing, and decoding (Chen K et al., 2020; Cao et al., 2022; Nguyen et al., 2021), as shown in Figure 1. Church et al. (2012) of Harvard Medical School stored 650 KB of data in DNA. The success of this experiment broke the notion that one could only use DNA to store a small number of bytes as in the early days. Moreover, this experiment stored data *in vitro* for the first time. This method realized the DNA storage of a larger amount of data and a practical application of DNA storage. Subsequently, the use of DNA to store data has become a hot topic in global research. Many research institutions have conducted research on DNA storage (Mathews et al., 2016; Organick et al., 2020). Grass et al. (2015) encoded an 83 KB file into 4,991 DNA fragments and then encapsulated each fragment with silica gel to finally achieve error-free data recovery. Blawat et al. (2016) developed an efficient and robust forward error correction scheme, which is suitable for DNA storage and can cope with errors in DNA synthesis, sequencing, replacement, *etc*. This encoding scheme demonstrates the viability of DNA as a long-term storage medium. Erlich and Zirlinsky (2017) used fountain codes to efficiently and concisely construct DNA encoding schemes. Their protocol generates varying numbers of oligonucleotides to achieve highly tunable redundancy without complicating the algorithm design. Erroneous oligonucleotides are removed during encoding, thus preserving high-quality sequencing fragments to ensure highly robust decoding. In their work, 2.15 MB of data was encoded into DNA sequences, and data recovery was realized. Song et al. (2018) proposed a scheme to convert binary sequences into DNA base sequences. The proposed encoding scheme not only achieved a high storage density of 1.9 bit/nt but also reduced the probability of base errors in the DNA sequence during synthesis and sequencing. Lopez et al. (2019) designed and validated an assembly strategy for DNA storage that can be extended to any DNA amplification process requiring nanopore sequencing. Zhang et al. (2020) developed an optimized Base64 method to achieve a high specific storage density of 1.77 bits/nt in DNA single strands. In this strategy, through Base64 encoding, code reconstruction and balance, and data mapping, some random text information was encoded into DNA sequences and the corresponding DNA molecules were synthesized. Then, they were inserted into circular plasmids for long-term information storage. The introduction of balanced codes in the transcoding process effectively controlled the GC content and homopolymers and reduced the error rate of encoding DNA synthesis and sequencing. The method enabled robust and efficient storage, and accurate readout of digital data.

Research has found that achieving random access of DNA sequences not only makes the DNA storage data scheme more functional but also reduces the cost of DNA storage data. Bornholt et al. (2016) described the architecture of a DNA-based archival storage system. The system maps keyword values to data functions and provides random access using common PCR amplification and primer identification. They also proposed a new coding scheme in the coding system that provides controlled redundancy. The feasibility, random accessibility, and robustness of the proposed coding were demonstrated through the synthesis of 151 and 42 KB data. Yazdi et al. (2017) first implemented a portable random-access platform using a nanopore sequencer. They designed an integrated processing pipeline that encodes the data to avoid costly synthesis and sorting errors, and enables random access *via* addressing. It uses efficient portable sorting through new iterations and includes the removal of error-correcting codes. They implemented a random-access DNA data storage system that used an error-prone nanopore sequencer but still produced error-free reads with the highest reported information rate/density. Therefore, their scheme represents, to a certain extent, a critical step in the practical application of DNA molecules as storage media. Organick et al. (2018) designed a large primer library that could individually read a particular file stored in the DNA. An algorithm was also developed to greatly reduce the coverage of sequencing reads required for error-free decoding by maximizing the information from all the sequence reads. Experiments demonstrated a feasible large-scale DNA data storage and retrieval system that utilizes the primer library to achieve random access to data. Tomek et al. (2019) used chemical processing in this scheme to selectively extract unique files from a complex DNA database simulating 5 TB of data. They designed and implemented a nested file address system that increased the theoretical maximum capacity of DNA storage systems by five orders of magnitude. This advancement enables the development and future expansion of DNA-based data storage systems with modern capacity and file access. Banal et al. (2021) encoded data as the sequences of DNA files encapsulated in silica capsules labeled with DNA barcodes on their surfaces. The scheme utilized Boolean logic operations to directly select barcodes to find files. It was demonstrated that image files were derived from a prototype 2 KB image database using fluorescence sorting, and the corresponding files were accessed. Banal et al. (2021) thus provided a scalable concept that can implement random access capabilities for large datasets in archive files.

In traditional DNA storage systems, DNA sequences are used to store data. Given that it is unrealistic to synthesize ultralong DNA sequences, files are divided into sub-blocks of fixed length, and each piece of data is stored in a short DNA sequence. As the storage of DNA sequences in the DNA pool is unordered, it is necessary to store the index (the position of the sub-block of the file in the file) into the DNA sequence so that the DNA sequence can be sequenced and decoded to restore the original file (Ceze et al., 2019). Li et al. (2018) proposed a DNA-based storage system that uses data concealment (steganography) to process addressing information. The idea is to embed the index into the DNA sequence corresponding to the data and use redundancy to embed the index in the data block. Hiding the index from sequences encoded by traditional schemes reduces unnecessary overhead in DNA storage systems. However, the encoding method using steganography has the problem that the index first needs to be restored from the data in the sequencing and decoding process to complete the sequencing and decoding.

To simplify the steganography decoding process and reduce the cost of the index in the DNA sequence, this article proposes using the hidden addressing method to process the index information for the DNA storage system and directly using the data instead of the index. The hidden addressing data can also directly participate in the decoding process. Compared with steganography to write indexes into data, the process of this solution is simpler and easier to operate. This article not only encodes the data using an encoding scheme of hidden addressing but also analyzes the local performance of the encoded sequence and the indexed overall self-similarity of hidden addressing in the results, and conducts experiments to simulate sequencing. The results showed that the variance of the GC content of the sequenced fragments was 0.004, indicating that the GC content of the coding sequence fragments of this scheme was relatively stable. The local thermodynamic properties of the sequences were better, which promoted the stability of DNA sequencing. The encoding sequence index was replaced by the data and the data were independent of each other, so the overall self-similarity of the index was low, which reduced the probability of errors in the DNA decoding process. The better sequencing effect proves the encoding performance to a certain extent.

The article is organized as follows. In chapter two, the article introduces encoding schemes with the hidden addressing properties and good sequence fragment performance. In chapter three, the results and analysis of the general encoding scheme evaluation indexes such as the overall self-similarity of the encoding scheme index, GC content of the sequence fragments, and net information density are presented. Finally, the fourth chapter contains the conclusion and suggestions for future work.

## 2 Encoding of Hidden Addressing

This chapter gives a detailed description of the DNA storage encoding scheme with the hidden addressing. This scheme not only achieves the characteristic of hidden addressing in the encoding process but also has the characteristic of a more stable thermodynamic property of the encoded sequence fragments. The specific flow and schematic are also given.

The pseudocode is shown in Algorithm 1. This scheme replaces the index with a few bits of data in the DNA sequence and suppresses the index of DNA sequence. First, the data are grouped so that each group of data is independent of each other, and then, each group of data is segmented to convert each segment of data into a sufficient amount of DNA fragments according to the fountain encoding method. This is because the fountain encoding method can generate a large number of DNA fragments that meet the conditions. In this scheme, each set of data will retain all fragments that satisfy the constraints to facilitate the subsequent selection of indexes from a large number of reserved shards. Therefore, one index DNA fragment and seven DNA fragments that can be decoded with the index are then selected from a large number of retained DNA fragments. Finally, the selected index DNA fragments and the DNA fragments decoded with the index are concatenated into a single DNA sequence output. This process is performed for each set of data until all data encoding is complete.

Algorithm 1 Encoding for DNA storage using data hiding addressing information.

**1** Divide the data into N groups, each group goes through such a process;
**2** then divide the data into n groups within the group;
**3 **generate Robust Solitary Distribution Functions from Segmented Data;
**4 **generate random seed → seed;
**5 **randomly select several pieces of data to XOR the data as $d_{i}$ ;
**6** $D_{i}$ =[seed $d_{i}$ ];
**7** $S_{i}=D_{i}\to \left\{A,T,G,C\right\}$ ;
**8 If** meeting the constraints
**9 **deposit S ;
**10 else** delete;
**11 end**
**12 end**
**13 for** each S-sequence
**14 **pick out the index sequence that satisfies the condition;
**15 end**
**16 return** final sequence;

### 2.1 Hidden Addresses and Indexes

Erlich and Zielinski (2017) proposed a fountain encoding scheme to construct DNA sequences through Luby transform encoding (Luby, 2002). The data are first grouped, then randomly XORed using a special robust isolation distribution function, and finally packed into many droplets. The droplets that did not meet the constraints were excluded. The droplets that met the constraints were used to synthesize oligonucleotide sequences, and sequencing decoding experiments were performed to achieve complete data recovery. In the fountain encoding scheme, Erlich and Zielinski (2017) used the seed as the index of the sequence to restore the original file. In this article, the method of group encoding is used to conduct fountain encoding in the group, and a hidden addressing method is used with blocks of data instead of indexes. This hides the index in the sequence. More detailed pseudocode for Algorithm 2 is shown.

Algorithm 2 Use data instead of addressing information.

**1** Each set of data corresponding to a large number of DNA fragments**;**
**2 for** N sets of DNA fragments with enough data
**3 **select a representative DNA fragment for each set of data**;**
**4 If** repeats of DNA fragments are selected in the i-th and j-th groups of data (where N> i > j)
**5 **select another DNA fragment from the j-th group of data**; end**
**6 If** the high similarity of the DNA fragments is selected in the i-th and j-th datasets (where N > i > j)
**7 **select another DNA fragment from the j-th group of data**;**
**8 end**
**9 end**
**10 return** record all selected indices representing the set of data**;**
**11 for** N sets of data, each set of data has enough DNA fragments
**12 **select 7 DNA fragments from each set of data**;**
**13 end**

**FIGURE 2:**
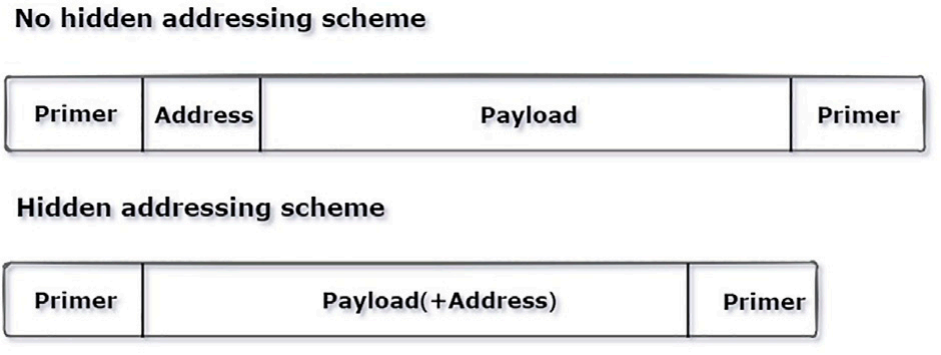
Comparison of the required sequence lengths with and without the hidden addressing scheme.

The index is hidden from the traditional encoding algorithm, which saves the cost of adding additional indexes. Figure 2 shows the number of bases required for encoding the same piece of data using the hidden addressing scheme and without the hidden addressing scheme. It is clear that the encoding scheme with hidden addressing requires fewer bases than the encoding scheme without the hidden addressing. In the DNA fountain encoding experiment, the author uses the “seed” as an identifier for sequence splicing. The seed is randomly generated, and the sequence similarity corresponding to some seeds is too high, which results in sequence splicing errors. However, in this study, the use of XOR data instead of an index makes the overall self-similarity of the hidden addressing “index” weaker, which can prevent the sequence from being too similar in the process of splicing and avoid sequence splicing errors.

### 2.2 Constraint Control of Sequence Fragments

#### 2.2.1 Restrictions

Errors such as substitution, insertion, and deletion of bases are prone to occur during the synthesis of DNA sequences during DNA storage and DNA sequencing (Wang et al., 2019). The error rate of each base in the DNA sequencing process is about 1% (Press et al., 2020; Zan et al., 2021). When there are some special sequences in the DNA sequence (GC content is high in the entire DNA sequence and existence of homopolymers of certain bases), this will easily lead to nonspecific hybridization of DNA during storage (Wang et al., 2019). Once the abovementioned hybridization reaction occurs in DNA, it will directly affect the normal progress of the DNA sequencing process, resulting in data read errors and read failures due to sequencing deviations (Chen Y. J et al., 2020; Zan et al., 2021). As this situation may easily cause instability of the DNA sequence, the sequence is generally required to comply with the constraints to reduce the incidence of nonspecific hybridization and reduce the error rate in the process of sequence reading and writing.

(1) The number of GC base pairs contained in the DNA has a great influence on the changes in melting temperature and free energy of DNA molecules. Therefore, under normal circumstances, the G and C content of a DNA sequence should be kept between 45% and 55% (Yin et al., 2021; Cao et al., 2022; Wu et al., 2022). The mathematical formula is as follows:

$$GC\left(content\right)=\frac{\left|G\right|+\left|C\right|}{\left|A\right|+\left|T\right|+\left|G\right|+\left|C\right|}\times 100\%.$$

(2) DNA sequences with longer homopolymer runs (DNA fragments of contiguous nucleotides or repeating bases) are prone to errors during synthesis, amplification, and sequencing (Ross et al., 2013). For example, in ACCCCAT, the presence of base repeats can easily be misinterpreted during sequencing as sequences such as ACCCAT. Therefore, it is necessary to limit the presence of three or more repeating bases in the DNA sequence. The mathematical formula for this is as follows:

$$S_{i}\neq S_{i+1}\neq S_{i+2},i\in \left[1,n-2\right].$$

#### 2.2.2 Sequence Fragments

A hidden addressing DNA storage coding scheme encodes the sequence. The procedure is as follows. First, group the data and then segment the data within the group. Each piece of data in the group is subjected to fountain coding XOR to convert each piece of data into a DNA sequence. Finally, the GC content and homopolymer constraints control the constraint filter to select the DNA that meets the constraints. Sequences that satisfy the constraints are retained, and those that do not satisfy the constraints are discarded directly. In this way, several short sequence fragments corresponding to each set of data are connected into a long sequence. The DNA sequence corresponding to each set of data satisfies the local GC content constraint and the homopolymer control constraint, which makes the sequence more stable than the sequence without local constraints control. The stability of the local GC content also ensures better local thermodynamic properties of the sequences. The local thermodynamic properties of the sequence are better, and the homopolymer control also ensures the sequence’s local stability and reduces the probability of errors during sequence sequencing.

### 2.3 Process and Schematic

**FIGURE 3:**
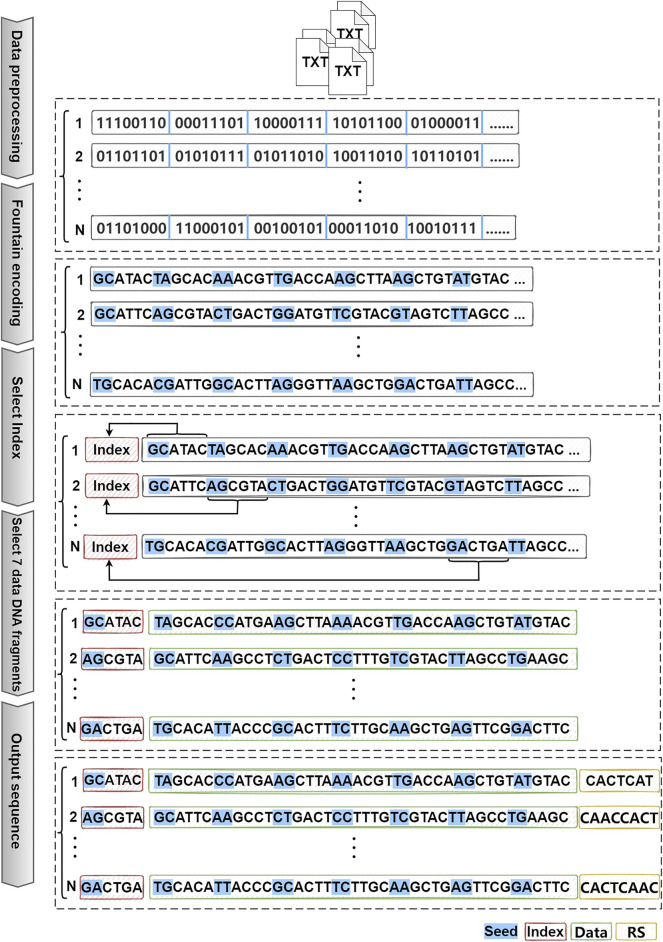
Schematic illustration of an example DNA storage encoding scheme for hidden addressing.

The overall schematic of the scheme is shown in Figure 3. It has three parts: file preprocessing, fountain encoding, and DNA fragment selection.

#### 2.3.1 Program-Specific Process

##### 2.3.1.1 File Preprocessing

The binary data are divided into N groups equally according to the size, and then the size of each group of data is divided into n segments. This allows each group of data to be independent of each other and makes it easy to read the data randomly. The segmented data fountain encoding control constraints in each set of data also make the GC content and homopolymer control of the encoding sequence fragments more stable.

##### 2.3.1.2 Fountain Encoding

First, distribution function Φ (Erlich and Zielinski, 2017) is generated according to the number of groups in the segment, and each time, a linear shift register is used to generate different random seeds. Second, d (where d∈[1,n] ) data segments are randomly selected to be XORed according to the distribution function. After the XOR, the seed is placed at the starting position of the XOR data, and then according to the {00, 01, 10, 11}→{A, C, G, T} method, seeds and data are converted into corresponding characters. Then, the converted string is filtered through the constraint filter, and in the filter, it is judged whether the sequence satisfies the constraints that it does not contain homopolymers and the GC content is kept at 45%–55%. Short sequence $S_{i}$ that satisfies the condition is temporarily saved, and short sequence $S_{i}$ that does not satisfy the constraint condition is directly discarded. Iteration is continued until the number of seeds is exhausted, and this set of data also produces a large number of strings $S_{1},S_{2},S_{3},...S_{\text{j}}$ that satisfy the constraints. In this step, N groups of data all go through such a process. Therefore, each set of data produces a corresponding $S_{1},S_{2},S_{3},...S_{\text{j}}$ .

##### 2.3.1.3 Select DNA Fragments

From the strings $S_{1},S_{2},S_{3},...S_{\text{j}}$ corresponding to each set of data, select $S_{k},k\in \left[1,j\right]$ representing the sequence as the index DNA fragments. If the index DNA fragments selected by the i-th sequence and the j-th sequence are the same or similar ( where,N>i>j ), another $S_{m},m\in \left[1,j\right],m\neq k$ is selected from the j-th sequence. After determining the index of the DNA sequence corresponding to each set of data, seven DNA fragments are selected from $S_{1},S_{2},S_{3},...S_{\text{j}}$ for each set of data. A total of seven DNA fragments in each set of data and the selected index can be decoded. In this way, index DNA fragment selected from each set of data is ligated with the seven DNA fragments. Finally, an RS error correction code is added at the end of each sequence for the error correction of the sequence.

#### 2.3.2 Scheme Example Diagram

Figure 3 shows a schematic diagram of a DNA storage encoding scheme for the hidden addressing. First, the TXT file is divided into N groups of data, and then each group of data is subjected to fountain coding, and each group generates a corresponding large number of DNA fragments. According to the similarity of the indexes, the DNA fragment sets corresponding to the N groups of data are horizontally compared, and the index DNA fragments of each group of data are selected. At the same time, seven DNA fragments were selected from each set of data and spliced with the selected index DNA fragments. Finally, RS error correction is added at the end of each sequence for the error correction after sequence sequencing.

In this section, based on the scheme of constructing high-efficiency DNA sequences with fountain codes, the hidden addressing scheme of this article is proposed, which is different from steganography, as data blocks are used to replace indexes in this scheme, whereas steganography embeds indexes into the data blocks. In the sorting phase, since the index is technically embedded into the sequence, the index is first solved from the data, and then processed through sorting, sequence assembly, and other operations. In this scheme, the sequencing can directly use the hidden addressing data blocks for sequence sorting, splicing, and other processes. Comparing the two, the process of this scheme is relatively simple and easy to decode. Based on the hidden addressing, it not only ensures that the local thermodynamic properties of the sequence are better but also uses the XOR data to replace the index, which reduces the overall self-similarity of the index and the incidence of sequence errors during splicing.

## 3 Results

To verify the performance of the index and sequence fragments of DNA sequences constructed by the hidden addressing DNA storage encoding scheme, this chapter compares and analyzes the Ehrlich and Zelinsky (2017) encoding scheme from two aspects: the overall self-similarity of the index and the constraint control analysis of the encoding sequence fragments by means of comparative experiments. The results show that the encoding experimental results of this scheme are better than Ehrlich and Zelinsky (2017) encoding experimental results. The net information density of the encoding scheme and its support for random access are also important indicators for evaluating the performance of the encoding scheme, which directly determine the scheme implementation cost. These are also compared in this chapter. The results show that the scheme has a high net information density and also has the functions of supporting random reading of files, decoding, and error correction. At the same time, the encoding sequence was also simulate-sequenced using the ART simulated sequencing tool.

### 3.1 Overall Self-Similarity

**FIGURE 4:**
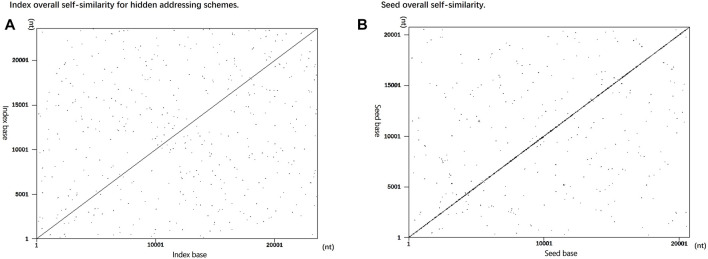
Self-similarity comparison between encoding indices. **(A)** Utilize data instead of overall self-similarity between indices. **(B)** Overall self-similarity between seeds used for splicing sequences in fountain coding experiments.

The overall index self-similarity of the encoding sequence is one of the important indicators to evaluate the encoding scheme. If the index similarity of the designed sequence is too high, it will lead to splicing errors after sequencing and affect the final decoding accuracy. Similarly, if the sequence similarity of the encoded payload is too high, the decoding accuracy will also be affected (Akhmetov et al., 2018). To verify the overall irrelevance of the sequence index design of the hidden addressing DNA storage encoding scheme, the self-similar visualization generator (Madeira et al., 2019) is used in this study to generate the resulting graph shown in Figure 4 for verification.

In Figure 4, the indices of the two storage schemes are used as input, and both use a word length of 10 as the input parameter for dot map generation. The encoding scheme using data-hiding addressing replaces the index with data blocks in the DNA sequence and omits the index from the DNA sequence. As the data are independent of each other, as shown in Figure 4A, there are not too many continuous repeating sequence fragments, indicating that the encoding sequence using the data-hidden addressing in this scheme does not have too many repetitions, which improves the accuracy of decoding. The sequence designed by the Erlich and Zielinski’s (2017) encoding scheme uses a seed as an identifier for sequence splicing. There will be two adjacent representation numbers between a single seed. After converting it into a DNA sequence, the similarity is high, so using the self-similarity visualization generator generates a graph showing that there will be a large number of concentrated and relatively continuous sequences. As shown in Figure 4B, there is a large number of concentrated repeated fragments in the sequence near the diagonal line, indicating that the seed used in this scheme as the sequence index has a large number of repeated short sequences.

In addition to using the Dottup dot plot generator to generate dot plots to visually compare the differences in overall self-similarity of coding scheme indices, the Jaccard similarity coefficient can also effectively calculate the overall similarity of the two encoding scheme index sequences and evaluate the overall self-similarity of encoding scheme indexes. The Jaccard similarity coefficient is a common way to calculate the similarity of two strings: The proportion of the number of elements in the intersection of two sets A and B in the union of A and B is called the Jaccard similarity coefficient of the two sets, and is represented by the symbol Jaccard(A,B) . The Jaccard coefficient is an indicator to measure the similarity of two strings. The higher the value, the higher the similarity between the two strings. On the contrary, the lower the similarity. The formula is as follows:

$$J\text{accard}\left(\mathrm{A,B}\right)=\frac{\left|A\cap B\right|}{\left|A\cup B\right|}=\frac{\left|A\cap B\right|}{\left|A\right|+\left|B\right|-\left|A\cap B\right|},\;A\neq \emptyset \;or\;B\neq \emptyset .$$

To calculate the Jaccard similarity coefficient for the two DNA sequences, we first transform each sequence into a set of k−mers . Let $S_{k}\left(q\right)$ be the k−mer set of sequence q containing the set of all contiguous subsequences of q length k . For example, the sequence $q_{1}=ACGTTAGGC$ , maps to the 5−mer set $S_{5}\left(q_{1}\right)\;=\left\{ACGTT,\;CGTTG,\;GTTAG,\;TTAGG,TAGGC\right\}$ For $q_{2}=\;GTACCTTAGG$ , sequence mapping to 5−mer set $S_{5}\left(q_{2}\right)=\left\{GTACC,TACCT,ACCTT,CCTTA,CTTAG,TTAGG\right\}$ , calculation of the Jaccard similarity coefficient of the two strings $q_{1},\;q_{2}$ is as follows:

$$J\text{accard}\left(q_{1},q_{2}\right)=\frac{\left|q_{1}\cap q_{2}\right|}{|q_{1}\cup q_{2}|}=\frac{\left|q_{1}\cap q_{2}\right|}{\left|q_{1}\right|+\left|q_{2}\right|-\left|q_{1}\cap q_{2}\right|}=\frac{1}{10}.$$

We compute the Jaccard similarity coefficients indexed in the two encoding schemes. In the two encoding schemes, each index sequence and the other sequences are divided by k−mers first, and then Jaccard calculation is performed and added to obtain a sum of the Jaccard similarity coefficients of each sequence. Finally, the Jaccard similarity coefficients of each sequence are summed up (Table 1).

**TABLE 1:** Jaccard similarity coefficient of two encoding schemes under different k−mers .

K-mers	6-mer	7-mer	8-mer	9-mer	10-mer	11-mer	12-mer
Our work	1,335.0	319.0	77.1	19.2	4.7	1.2	0.3
Erlich and Zielinski (2017)	1,435.5	480.7	223.1	133.3	85.3	53.5	22.2

The differences of hidden addressing indexes are analyzed through the visual analysis and quantitative analysis. This result shows that the index of the encoding scheme proposed in this study is better than the seed used by the encoding scheme of Ehrlich and Zelinsky (2017).

**FIGURE 5:**
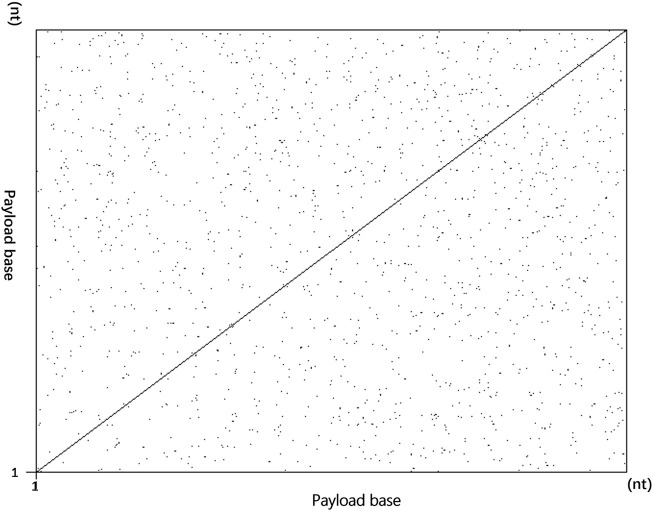
Overall self-similarity generated dot plot of the encoded payload sequence.

In addition to the comparative experiments on the overall self-similarity between the encoding scheme sequence indices, the overall experiments on self-similarity were carried out on DNA sequence encoding payloads. As shown in Figure 5, the input of this figure is the sequence of the scheme encoding payload. Using a word length of 12 as a parameter, there is no obvious long-sequence repetition in the figure. The repetition of individual short sequences has little effect on the final sequencing result (Akhmetov et al., 2018).

### 3.2 Code Fragment Performance Analysis

**FIGURE 6:**
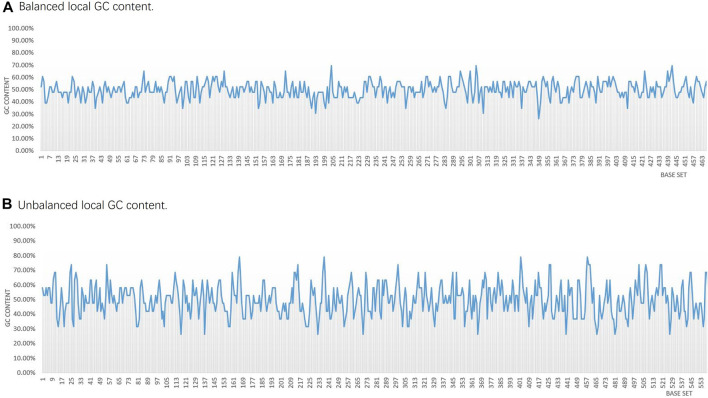
Statistical comparison of partial local GC base content in the two DNA storage systems. **(A)** This scheme carries out the local GC content control. **(B)** Fountain coding scheme without the local GC content control.

When reading the DNA storage data, the DNA sequence needs to be sequenced. For example, the sequencing-by-synthesis method is used in sequencing on the Illumina platform. In this process, for the continuously extended sequence fragments, satisfying the local GC content and homopolymer control constraints will make the sequencing fragments more stable, and the more stable sequencing fragments will also improve the sequence accuracy during the sequencing process (Akhmetov et al., 2018). To analyze the performance of sequence fragments in a DNA storage encoding scheme with hidden addressing, a statistical analysis of the GC content in the encoding sequence fragments was performed, as shown in Figure 6.

The scheme first divides the data into groups and segments, and then encodes the data into DNA fragments according to the fountain encoding conversion method. Each generated DNA fragment is added to a constrained screening process, and finally, the encoded DNA fragments are spliced into a nucleotide sequence. As shown in Figure 6A, most sequences can guarantee a GC content of 40%–60%, with a GC content variance of 0.004. It can be seen that the fluctuation range is small, and each fragment can satisfy the GC content balance constraint and the homogeneity polymer control constraint, which ensures that the local thermodynamic properties of the coding sequence are more stable. In the case of encoding the same txt file, the sequences encoded by the fountain encoding scheme in the Ehrlich and Zelinsky (2017) encoding scheme only conduct the overall GC content balance constraint of the sequence but not the local GC content balance constraint. Therefore, the local GC content of most sequences is unevenly distributed, and the variance of GC content is 0.011, which shows that the fluctuation range is large, as shown in Figure 6B. The length of each sequence encoded by the encoding scheme in Figure 6A is 184 bpl, so the GC content of each sequence is calculated as every 23 bases. In the sequences encoded by the encoding scheme in Figure 6B, the length of each sequence is 152 bp, so the GC content of each sequence is calculated as every 19 bases.

### 3.3 Encoding Performance

By evaluating the general characteristics of the encoding scheme and comparing it with the previous research results, we found that the DNA storage coding scheme with hidden addressing also has a high net information density, supports random access to files, and has error correction properties (Table 2).

**TABLE 2:** Comparison of existing encoding schemes.

Method	NID	RA	DS	EC
Church et al. (2012)	0.83	No	650 KB	Yes
Goldman et al. (2013)	0.33	No	739 KB	Yes
Grass et al. (2015)	1.14	No	83 KB	Yes
Bornholt et al. (2016)	0.88	Yes	15 KB	Yes
Blawat et al. (2016)	0.92	No	22 MB	No
Erlich and Zielinski (2017)	1.57	No	2.14 MB	Yes
Dimopoulou et al. (2021)	1.31	Yes	260 MB	No
Our work	1.48	Yes	10.1 MB/40 KB	Yes

In the encoding scheme of DNA storage, the evaluation indicators such as net information density (NID), random access reading support (RA), and support for error correction (EC) are important references for evaluating the encoding scheme. Net information density is also an important evaluation indicator to measure an encoding scheme, and its size directly determines the cost of DNA storage in the synthesis, sequencing, and error correction. Although the net information density is 1.48 nt/bit in this scheme, this scheme realizes the random access function of the DNA storage system. There are eight seeds used for decoding in each sequence so that each group of data is encoded separately to eliminate the integrity of the data in the fountain encoding experiment. Therefore, the redundancy in each sequence accounts for 26%, and the calculation formula is as follows

Redundancy=seed(5nt)×8+RS(8nt)Sequence length(184nt)×100%.

Implementing random access to files in an encoding scheme is also an important aspect to evaluate the performance of an encoding scheme. In the DNA fountain encoding experiment, there is a disadvantage of losing random readability. For example, if file F1 is required, the DNA sequences of all the stored data in the pool must be read to obtain required file F1. As DNA sequencing can take longer time and cost more than reading data from a hard drive, the encoding scheme designed in this study takes into account random read operation of files in the DNA storage system. Each file corresponds to several sequences with hidden addressing. If one wants to read file F1 from the DNA storage system, one only needs to read the sequence of the hidden addressing data block corresponding to F1 to realize the random reading of the file. This improves the random readability of the fountain encoding scheme devised by Ehrlich and Zelinsky (2017).

### 3.4 Sequence Decoding

To further validate the DNA encoding scheme using cryptic addressing, we performed relevant simulate sequencing experiments on the encoded data using simulate sequencing tools.

ART is an analog tool for the next-generation sequencing reads (Huang et al., 2012). Simulated sequencing readings are generated by simulating the sequencing process using built-in, technology-specific read error models and baseline value profiles that are empirically parameterized in large sequencing datasets. All three major commercial next-generation sequencing platforms are currently supported: Roche’s 454, Illumina’s Solexa, and Applied Biosystems’ SOLiD. We used ART Illumina simulate sequencing with encoding sequences directly as input, reads were mock single-ended, 150 bp in length, the maximum total number of inserts and deletes per read is set to 0, 20 × coverage, and the Illumina sequencing system profile was MiSeq v1 (250 bp). The results showed that more than 95% of the bases in each sequence were detected by simulated sequencing.

After the N sequences in the pool are measured, the DNA sequence is corrected according to RS error correction. The error-corrected sequence is then fed into the file preprocessing system, which automatically identifies the data used to hide the index. The sequences are then sorted according to the reference ranking table (each encoded data has a reference ranking table, as shown in Supplementary Table S1. In this experiment, due to the large number of sequences, the reference sorting table encoding the 40 KB data is only partially shown in the Supplementary Material; the rest will be shown on Github: https://github.com/wangpenghaoAA/Reference-order). Input the sorted sequence into the decoding program in order. Each sequence is converted to binary according to the conversion rule {A, T, G, C}→{00, 11, 01, 10} . Each of the N groups of data can decode the eight pieces of short data according to the seed, and restore the inverse process of XOR (detailed code: https://github.com/wangpenghaoAA/Fountain_imp). Finally, the decoded data are spliced to restore the original data.

In this chapter, the hidden addressing DNA storage encoding scheme proposed in this article was analyzed from the overall self-similarity of the index, the performance of the encoding sequence fragment, the general evaluation index of the encoding scheme, and the related simulated sequencing experiments. The results show that this scheme not only has a lower similarity between the data using hidden addressing but also has better local thermodynamic properties of the coding sequence, support for random reading of files, support for file error correction, higher net information density, and better simulation sorting results.

## 4 Conclusion

In this study, a hidden addressing DNA storage encoding scheme was proposed, which is based on the fountain encoding construction of DNA sequences. This encoding scheme uses data-hidden sequence addressing. Using data instead of indexes in this scheme not only saves indexes and reduces costs but also makes it easier to operate than steganography. This study also analyzes the overall self-similarity of coding sequence indices through the Jaccard similarity coefficient and the Dottup dot plot generator. The index of this scheme is replaced by data, and the data are independent of each other and have low correlation. Therefore, the index replaced by the independent data will reduce the overall self-similarity of the index, and the encoding scheme with lower overall self-similarity of the index will help to avoid splicing errors. At the same time, this study also analyzed the GC content of the coding sequence fragments. The data were first grouped; each group was segmented; and finally, they were encoded into a scheme of DNA sequences. The GC content and homopolymer control constraints were satisfied when encoding each data fragment. Therefore, the local thermodynamic properties of the sequences were better, which increased the local stability of the sequences. Finally, we also compared the general evaluation indicators of the encoded data and performed related simulated sequencing experiments. The results showed that the proposed DNA storage scheme has the advantages of higher net information density, support for random access, support for error correction, and better sequencing results.

Current DNA storage and error correction schemes are fault-tolerant to synthesis and sequencing errors, and even the loss of complete sequences. If the encoding and error correction strategies can be further optimized, DNA-based storage can be implemented using low-cost and low-fidelity technology (Cai et al., 2021). In our future work, we will continue to work on DNA storage with a focus on DNA coding technology and DNA sequencing technology. We believe that there is a lot of room for improvement in this developmental path. A large amount of work has been done, such as a comprehensive evaluation of DNA storage error models. The future of synthetic DNA storage systems remains bright and could have profound implications for areas such as global data management and healthcare. With the joint efforts of academia and industry, there will be many ways to build low-cost and practical DNA storage schemes in the future.

## Data Availability

The original contributions presented in the study are included in the article/Supplementary Material. Further inquiries can be directed to the corresponding author.
