# Supplementary material for: Hidden Addressing Encoding for DNA Storage
Source: Front Bioeng Biotechnol. 2022 Jul 19;10:916615. doi: 10.3389/fbioe.2022.916615 (PMC9344065; doi:10.3389/fbioe.2022.916615)
Supplement: Supplementary file 1 [file DataSheet1.PDF]

Supplementary Material

1 Additional table

TABLE 1 | Sequence reference for sequenced sequences.

Serial number	Index number	Serial number	Index number
1	12554019835070	13	848763086260
2	7228970999289	14	4888818944578
3	121558437221	15	15444627855172
4	10492258180879	16	16151735755247
5	10427167494980	17	12832049053867
6	9862216397547	18	16456554095806
7	1293095265576	19	953756111131
8	12775796364059	20	4861352363851
9	3349027052538	21	16153412389812
10	1478694400385	22	1480659235565
11	4936261283987	23	14677954247995
12	8512915544879	24	7091029102363

Too many sequences, see the full version: https://github.com/wangpenghaoAA/Reference-order.

2 Additional information

1. Seeds are used in both this scheme and the fountain encoding experiment. What is the difference between the seeds used in the two schemes?

In fact, the seeds used by the two schemes are essentially the same for ease of decoding. But the seeds in the fountain encoding experiments [1] had another role. That is feature recognition for sequences, but at the same time exposes a problem. When the DNA sequences of the seeds of the two sequences are too similar, it is easy to cause sequence decoding errors. And fountain encoding is to XOR between N groups of data to generate DNA sequences that meet the constraints. When a sequence is decoded incorrectly or lost, it is bound to result in the loss of the entire data. As shown in Figure 1:

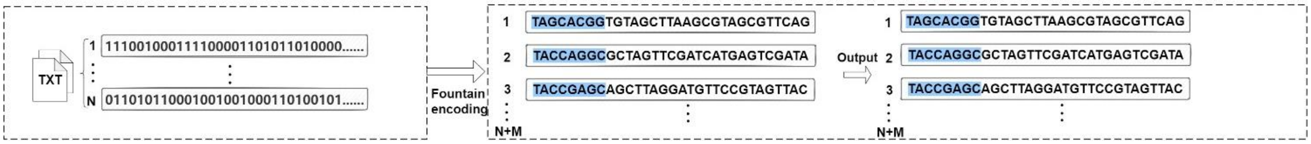

FIGURE1|Schematic diagram of the fountain encoding experiment.

In our scheme, we divide the data into N groups and then perform fountain encoding on sub-segments of each group of data. Therefore, the N groups are independent of each other and do not affect each other. When each set of data is fountain encoded, a large number of seeds and XOR data are converted into DNA fragments, so each set of data will generate a large number of DNA fragments that meet the constraints. From the generated large number of DNA fragments that meet the constraints, we select the index DNA fragments of this set of data, and 7 DNA fragments that can be decoded with the index. After similarity search, we will select DNA fragments with low similarity as indexes, so we can avoid errors caused by the high similarity of seeds in the fountain encoding experiment. In our scheme the seed is used in decoding each set of data without feature recognition. As shown in Figure 2:

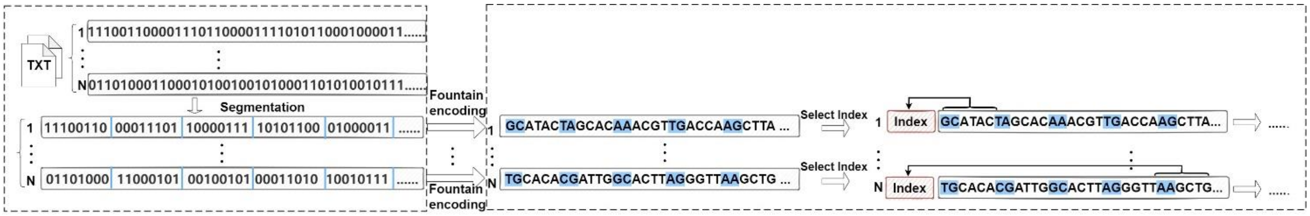

FIGURE2|Schematic diagram of an encoding scheme for hidden addressing.

2. Hidden addressing details：

Hidden addressing is not to discard the index directly, but to replace it with data block. In other words, the index exists in the DNA sequence in the form of data block, and the data block that replaces the index has to assume the role of the data block and the role of the index in the DNA sequence. We have done two things about your question about hiding addressing details: ① as shown in the following figure 3:

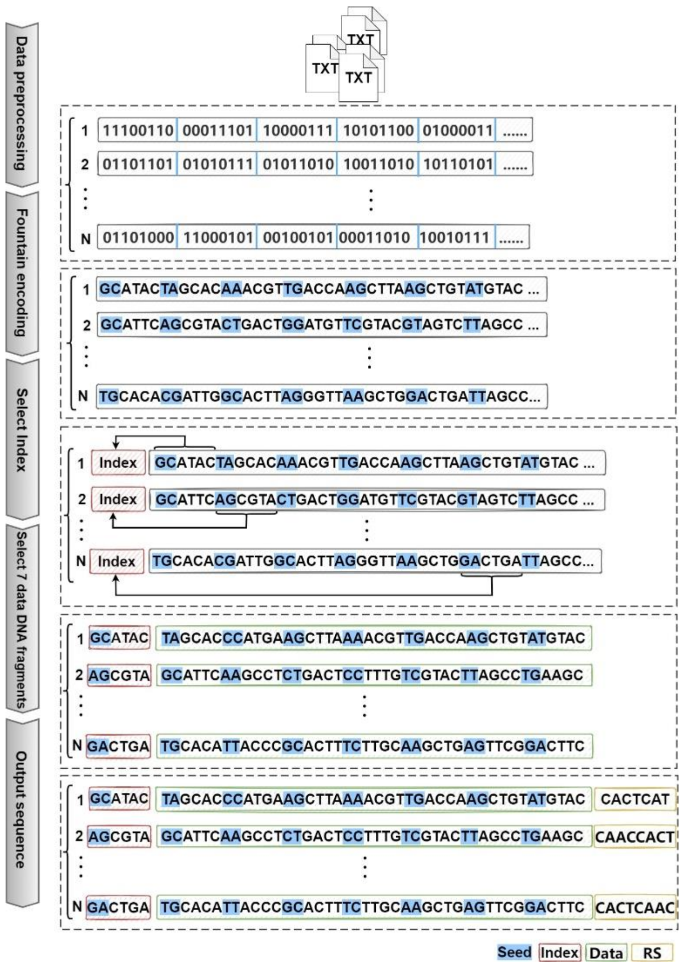

FIGURE 3|Schematic of hidden addressing

② Pseudocode for addressing using data hiding:

Algorithm 1. Use data instead of addressing information

```
Each set of data corresponding to a large number of DNA fragments;
for  N sets of DNA fragments with enough data
        select a representative DNA fragment for each set of data;
        If repeats of DNA fragments selected in the i-th and j-th groups
            of data (where N> i > j)
            select another DNA fragment from the j-th group of data;
        end
        If the high similarity of the DNA fragments selected in the i-th
            and j-th data sets (where N > i > j)
        select another DNA fragment from the j-th group of data;
        end
end
return  record all selected indices representing the set of data;
for  N sets of data, each set of data has enough DNA fragments
        select 7 DNA fragments from each set of data;
end
```

3. The net information density can theoretically be improved after hidden addressing, but in fact, the net information density does not change significantly.

In the fountain encoding experiment[1], the author failed to achieve random reading of data, and directly XORed N groups of data. If a sequence loss is encountered, it may cause the file to fail to decode. In our encoding scheme, not only hidden addressing is achieved, but also the data sets are independent of each other, allowing the implementation of random-access functions. Each group of data is independent of each other and does not affect each other. When one sequence is lost, the decoding of other sequences is still unaffected and can be less lost within the maximum range. Although data is used instead of indexes to reduce redundancy in this paper, higher encoding density is obtained relative to other storage systems that implement random access functions. Although the 8 seeds added by the fountain encoding for each set of data during the encoding process will result in a lower storage density (1.48nt/bit) than that of the fountain encoding experiment (1.57nt/bit)[1], it is worth mentioning Erich's encoding scheme random-access is not implemented. Therefore, we believe that it is fair to compare the indicators of storage systems that implement the same function, so the relevant comparisons are made below.

For example, in 2018, Organick[2] designed a large primer library that could individually read a particular file stored in DNA. Experiments demonstrated a feasible large-scale DNA data storage and retrieval system that utilizes the primer library to achieve random access to data. In 2019, Tomek[3] designed and implemented a nested file address system that increased the theoretical maximum capacity of DNA storage systems by five orders of magnitude. This advancement enables the development and future expansion of DNA-based data storage systems with modern capacity and file access.

This is a representative scheme that can realize random reading of files at present. The comparison results are shown in Table 2:

TABLE 2|Net information density comparison of random-access functions.

Method	Net information density	Random access	Data size
Erlich[1]	1.57	No	2.14MB
Organick[2]	1.10	Yes	200.2MB/32KB
Tomek[3]	0.66	Yes	94.3KB
Our work	**1.48**	**Yes**	10.1MB/40KB

3 Reference

1. Erlich, Y. and D. Zielinski, *DNA Fountain enables a robust and efficient storage architecture.* Science, 2017. **355**(6328): p. 950-953.
2. Organick, L., et al., *Random access in large-scale DNA data storage.* Nature Biotechnology, 2018. **36**(3): p. 242-+.
3. Tomek, K.J., et al., *Driving the Scalability of DNA-Based Information Storage Systems.* ACS Synthetic Biology, 2019. **8**(6): p. 1241-1248.
